# A review of symptom, pathogenesis and treatment characteristics of the elderly with chronic insomnia

**DOI:** 10.1097/MD.0000000000041346

**Published:** 2025-01-31

**Authors:** LiBo Xia, HaiYan Liu, JiXiang Ren

**Affiliations:** a Department of General Medicine, Affiliated Hospital of Changchun University of Traditional Chinese Medicine, Changchun, China; b Department of Medical Section, Changchun Second Hospital, Changchun, China; c Affiliated Hospital of Changchun University of Traditional Chinese Medicine, Changchun, China.

**Keywords:** chronic insomnia, circadian rhythms, Sen citizen, sleep structure

## Abstract

One third of a person’s life is in a state of sleep, and good sleep quality is one of the indicators of health. Long-term insomnia has a great impact on patients’ quality of life, physical and mental state. Especially in the elderly, long-term insomnia will induce a variety of chronic diseases, seriously affecting the quality of life. Because of the characteristics of the physiological structure of the elderly, insomnia in the elderly has its unique characteristics different from that of the young. This article reviews the characteristics of chronic insomnia in the elderly in terms of symptom, pathogenesis and treatment. By elaborating the characteristics of senile chronic insomnia, we hope to provide ideas for clinical treatment of senile chronic insomnia.

## 1. Introduction

Elderly chronic insomnia is aged more than 60 years, starting difficulties, maintain them or non restorative sleep sleep, continue for at least 3 months, and cause significant distress and daytime function in patients with damage of the disease. The incidence of insomnia in various countries around the world is increasing year by year, according to relevant statistics: the total incidence of about 15.0%.^[1]^ Incidence and each age appear significantly different, the prevalence of insomnia in the elderly was obviously higher than that of middle-aged and young people,^[2]^ the prevalence of insomnia in the elderly was 50%,^[3]^ that is to say, every 2 old people, there is a person insomnia problems.

Insomnia is a lot of disease cause, and the occurrence of these diseases and affects the patient’s quality of sleep, form a vicious circle, and then increase the degree of insomnia and related diseases. Especially for the elderly, long-term insomnia can induce hypertension, heart disease, diabetes and neurodegenerative diseases.^[4–6]^ Study found that the incubation period is more than 30 minutes of sleep, the sleep efficiency is lower than 80% or rapid eye movement sleep (REM) higher than the total long when a 25% increased risk of death in the elderly.^[7]^

## 2. Symptom characteristics of chronic insomnia in the elderly

Compared with insomnia in young people, insomnia in the elderly has its unique characteristics, including changes in circadian rhythm and sleep structure, due to degenerative changes in organs and tissues with aging.

### 2.1. Changes in circadian rhythm

Circadian rhythms regulate physiological and behavioral processes in the human body.^[8]^ The biological clock that regulates circadian rhythms is dynamic and evolves over time. Importantly, the patterns of sleep and wakefulness undergo significant changes throughout various stages of life. In the human body, the suprachiasmatic nucleus (SCN)^[9]^ is the circadian clock that dominates the circadian rhythm. The SCN can receive light signals that integrate the external environment, and then transmit the time information of the day to various brain regions through synapses and diffusible signals, so as to synchronize the rhythmic activity of the whole body with the light-dark cycle of nature. The circadian clock includes a core set of genes that interact through a series of negative feedback pathways.^[10,11]^

#### 2.1.1. Circadian change of rest – activity

In real life, the most easily observed characteristics of circadian rhythms are the preference for going to bed early or late, and getting up early or getting up late. In humans, the observed age-related circadian shift is a preference for early sleep versus early rise. The results of a cross-sectional study comparing the responses of older and younger adults to sleep type assessment tools suggest^[12]^ that adults aged 60 years and older are more likely to identify with the trend of early wake-up and early bedtime than adults aged 20 to 40 years. At present, there is a lack of longitudinal studies on individuals to track the changes in their circadian rhythm with age, but existing research supports the results of cross-sectional design.^[13,14]^ Broms^[15]^ conducted a longitudinal tracking study on 567 adults for 23 years and found that their rest activity rhythm gradually shifted to the “early to bed and early to rise” pattern. Retrospective self-comparative studies of older participants have also shown a tendency to become “early to bed, early to rise” as they age.^[16]^

#### 2.1.2. circadian change of sleep – wake

**Figure 1.**
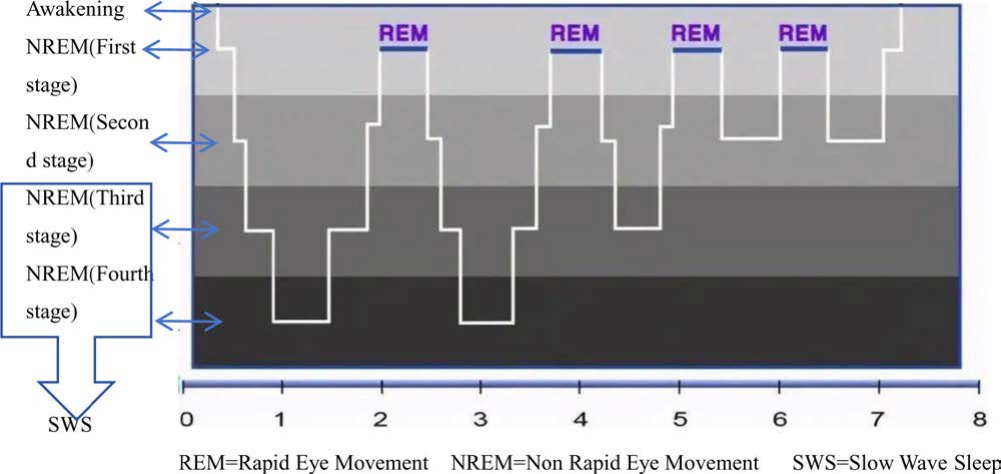
8-hour sleep cycle under normal physiological conditions.

The timing and structure of sleep undergo many changes with aging. Study found that older people (average age 68), on average, than young people (average age was 23 years old) sleeping time^[17]^ 1 to 2 hours in advance. Compared with younger adults, older adults experience more fragmented sleep, have longer latency to fall asleep, and spend less time in stages 3, 4 and REM sleep.^[18]^ This fragmented sleep has also been found in other species, such as old monkeys, old hamsters, and old fruit flies.^[19–21]^ VanCauterE^[22]^ found that from the age of 40, the total amount of sleep decreased by about 30 minutes for every 10-year increase in age. Sleep fragmentation poses challenges for older adults in falling asleep at night and diminishes their daytime activity, potentially impacting their overall quality of life (Fig. 1).

#### 2.1.3. Circadian change of core body temperature

When core body temperature drops, the body will feel sleepy, and when core body temperature rises, it is easy to wake up. In young adults, core body temperature rhythms typically peak in the late afternoon and nadir in the early morning.^[23]^ In the elderly, although the period of this rhythm remains stable^[24]^; However, research has shown that the amplitude of the circadian rhythm in older people is reduced by 20% to 40%, with the lowest core body temperature being higher than that of young people.^[25]^ Research has also indicated that the phase of this rhythm tends to advance in older individuals.^[26]^ Insomnia at night in the elderly may be associated with a slightly higher nadir of core body temperature than in the young, and the forward shift of sleep phase may be related to the forward shift of circadian rhythm of core body temperature. Circadian changes in sleep coincided with circadian trends in core body temperature.

#### 2.1.4. Circadian changes in melatonin secretion

Melatonin is a hormone secreted by the pineal gland, the secretion has obvious circadian rhythm, secretion is suppressed during the day, secrete active at night. Melatonin plays a crucial role in synchronizing the body’s internal clock by regulating core body temperature and gently promoting sleep.^[27]^ Studies in human evidence^[28]^: melatonin secretion decline with the growth of the age, total compared with young people, the elderly melatonin secretion peak appeared at night time earlier, but the total reduction. WallerKL reported^[29]^ that older adults who developed cognitive impairment had lower nocturnal melatonin peaks compared with healthy controls. In addition, in patients with neurodegenerative disorders or a prodrome of individuals, melatonin synthesis and melatonin receptor expression significantly reduce.^[30]^

### 2.2. Changes in sleep structure

#### 2.2.1. Overall changes in sleep structure

Insomnia in young people is characterized by difficulty falling asleep, mostly due to stress or emotional factors. However, if there is a good sleep environment, the total sleep duration is unchanged, and most of them do not affect their normal work and study during the day. Compared with insomnia in the young, insomnia in the elderly has the following characteristics^[31–34]^: The earlier sleep time; Longer sleep onset latency; Shorter overall sleep duration; More fragmented sleep; More fragile sleep; Awake all night time increased; and Daytime sleepiness.

#### 2.2.2. Microscopic changes of sleep structure

##### 2.2.2.1. Decreased slow-wave sleep

Slow-wave sleep (SWS) is important for restoring energy and physical strength. SWS is characterized by synchronous slow wave activity (SWA) on EEG, and the decrease of SWA in EEG indicates the decrease of SWS. We have observed a significant reduction in SWA in middle-aged people compared with young people, and this impairment in SWA is particularly prominent in the elderly.^[35]^ Interestingly, the study revealed that the age-related decline in SWA was not uniformly diminished across each non rapid eye movement (NREM) sleep cycle. In contrast, the greatest age-related absolute SWA reduction was seen during the first NREM sleep cycle, with an average absolute SWA reduction of 75% to 80% compared to young adults.^[36]^

SWA is intricately linked to sleep stability. As an individual remains awake for extended periods, sleep pressure accumulates, resulting in increased SWA during subsequent sleep. Among young individuals, SWA peaks during the initial NREM cycle of normal sleep and subsequently declines exponentially in the following NREM cycles.^[35]^ This homeostatic sleep regulation process also changes with age, and the exponential slope of nocturnal SWA dissipation is shallower in the elderly than in the young, indicating that the steady-state discharge decreases with age and the steady-state regulation of SWA is impaired in the elderly.^[37]^

The observed changes in SWA are attributed to a marked reduction in both the amplitude and density of slow waves during middle age, with further declines in amplitude and frequency of slow waves continuing as individuals advance in age.^[38]^ In comparison to younger individuals, the average frequency of slow waves observed in older adults is approximately 0.1 Hz lower.^[39]^

##### 2.2.2.2. Decreased sleep spindles

Sleep spindles are produced by the thalamus, reflecting transient undulating oscillatory activity in the range of 12 to 15 Hz, and their role is to prevent external environmental noise from disturbing the sleeping brain.

Compared with young people, old people sleep spindles frequency decreases.^[40]^ Lowering the frequency of sleep spindles and age related to a certain extent related to the decrease in the number of sleep spindles produced. As the growth of the age, unit time the number of sleep spindles or a significant reduction in the density of,^[41]^ unique characteristics of the spindle will be affected by similar, these seem to have led to the signal power associated with sleep spindles of the overall decline, for example, compared with the young, the elderly of the duration of sleep spindles, peak and average amplitude decrease.^[42]^

## 3. Etiological characteristics of chronic insomnia in the elderly

Elderly chronic insomnia numerous causes, including: changes in physiology, pathological factors, drug use, and substance abuse, social psychological factors.

### 3.1. Changes in physiological structure

#### 3.1.1. SCN structural changes

A cross-sectional study showed that the SCN volume of old people was smaller than that of young people, and the total number of SCN cells was lower in old people.^[43]^ The amplitude of firing rhythms in SCN neurons is significantly lower when compared to that observed in younger individuals.^[44]^ SCN gamma-aminobutyric acid (GABA) can synapses lost along with the age growth, so the older animals less inhibition of GABA can stimulate the SCN neurons also.^[45]^ More units in the rodents record shows that as the growth of the age and the activity of SCN amplitude is reduced, and the lower older animals may reflect the SCN cells firing pattern of the consistency of the loss of.^[46]^

#### 3.1.2. Structural changes of the crystalline lens

Zeitgeries are substances that coordinate the circadian rhythm in an organism with the 24-hour rhythm of the external environment, so reduced sensitivity to environmental zeitgeries in the elderly may lead to circadian changes in later life. Light is the most powerful zeitgeber. There is no significant difference in the total amount of light exposure that individuals receive throughout the day; however, there may be critical differences in the manner in which light is transmitted through the eyes of younger and older adults. As individuals age, the crystalline lens of the eye undergoes changes, becoming both yellowed and thicker, which results in a 1% reduction in the total light passing through the retina per year in people over the age of 18.^[47]^ This degenerative change results in an increased selectivity of the lens for shorter wavelengths of light, which are preferentially absorbed by light-sensitive retinal ganglion cells,^[48]^ and in older adults, the degree of crystalline lens yelosis is positively correlated with sleep disturbances.^[49]^ Cataracts are also associated with impaired sleep quality, and cataract extraction can have beneficial effects on sleep efficiency and the amplitude of melatonin rhythms in the elderly,^[50]^ with the effects is particularly evident in populations with extremely poor sleep quality.^[51]^

#### 3.1.3. Structural changes of sleep-wake nuclei

Age-related sleep structure and the change of phase maintain system abnormalities associated with sleep - wake, which result in the instability of sleep. The mechanisms of stable sleep and arousal originate from the synergistic action among brainstem, midbrain, and hypothalamic nuclei.^[52]^ Certain neurons within the hypothalamic prefrontal region are capable of expressing the inhibitory neuropeptide galanin, which plays a crucial role in initiating and sustaining sleep.^[53]^ The number of neurons that express ghrelin significantly decreases with age, and this decline is proportional to the severity of sleep fragmentation observed in older adults.^[54]^

Uplink awakening system to promote wake brain stem nuclei and the lateral hypothalamic area to promote wake orexins expressed the awakening of neurons to help maintain stable state,^[55]^ also experienced age-related deterioration. In rodent models, compared with the young adult rats, senile rats express orexins neurons decreased 40%.^[56]^ In human autopsy study, the effect of small amount, but it is still significant, observed in the older population reduced 10%.^[57]^ Greater effects were observed in patients with insomnia accompanied by cognitive impairment, suggesting that a substantial loss of orexin-expressing neurons may be responsible for worsening sleep-wake instability in this context.^[58,59]^

In addition to alterations in sleep-wake regulation, the decrease in nocturnal sleep among older adults is also correlated with a reduction in gray matter thickness.In a study, a correlation was observed between poor sleep quality in older adults and a reduction in gray matter volume^[60]^ and further predicted the severity of sleep fragmentation. Sleep quality worse and thinning of the gray matter which is the cause, which is the fruit has not been determined, in spite of this, the structure and function in age-related changes will inevitably lead to the deterioration of regulating sleep and wake.

It is important to note that not all sleep and wake up adjustment nuclei is influenced by age, it shows that the certain specificity. For example, in human autopsy, serotonin-expressing neurons in the dorsal raphe nucleus^[61]^ and histamine-expressing neurons in the hypothalamic tuberotemial nucleus^[62]^ show small age-related changes.

#### 3.1.4. reduced susceptibility adenosine receptors

Sleep - wake up regulating system controlled by circadian rhythms and homeostasis in the body system, ideally, these 2 systems together cause drowsiness, prompting we have high quality and enough sleep.^[63]^ The function of the homeostatic system is to restore the body to a state of rest, and it is affected by a variety of neurotransmitters, the most important of which is adenosine, which is a metabolic byproduct. The longer the waking time, the more adenosine is accumulated, and the more drowsiness will be produced.^[64]^

It was found that extracellular adenosine levels appeared to be higher in aged rodents than in young rodents. These age-related differences in the brainstem and the basal forebrain sleep - wake especially regulation center.^[65]^ This finding appears to contradict the general observation derived from the aforementioned human aging study, which indicates a lower steady-state sleep drive. While elevated adenosine levels typically suggest a stronger steady-state drive for sleep, this particular case exhibited a reduced steady-state drive to sleep.

A mechanism is to reconcile the difference with age exist in the cortex, hypothalamus, hippocampus and striatum region of adenosine A1 receptors widely loss^[66]^ obviously, along with A1 receptor gene expression.^[67]^ Thus, although increased adenosine levels reflect higher sleep demands in older adults, decreased A1 receptors may decrease sensitivity to higher extracellular adenosine concentrations. Consequently, the attenuation of receptor sensitivity inhibits a higher steady-state pressure from resulting in an increased stable sleep drive, thereby precluding any extension of SWS and SWA.

However, changes in adenosine alone are unlikely to fully account for age-related changes in steady-state sleep-driven expression. Other theories include possible regulation of adenosine A1 receptor-dependent signaling and age-related changes in glial function.^[68,69]^

#### 3.1.5. Abnormal secretion of excitatory neuropeptide-- orexin

Insomnia is an excessive arousal disorder, in which abnormal secretion of a certain neurotransmitter leads to sustained excitation of brain neurons without inhibition.

The orexin system is essential for maintaining wakefulness and regulating the transition between sleep and wakefulness.^[70]^ Orexin activates the cerebral cortex and stimulates cholinergic neurons in the basal forebrain (BF),^[71]^ thereby promoting arousal. The injection of orexin into the BF has been shown to effectively enhance arousal states while reducing NREM.^[72]^ This phenomenon can be attributed to the interaction between orexin neurons and cholinergic neurons in the BF, which leads to depolarization of the latter – a process that plays a crucial role in regulating arousal. Orexin neurons exhibit a dense projection to the locus coeruleus noradrenergic (NE) neurons, thereby regulating their activity and influencing appetite. NE neurons demonstrate a specific firing pattern: their firing frequency is highest during wakefulness, decreases during NREM sleep, and is negligible during REM sleep.^[73]^ Consequently, orexin indirectly modulates NE neuron activity to regulate wakefulness.

Elderly people have more sensitive appetite hormone neurons,^[74]^ which are closer to the discharge threshold and can secrete a large amount of appetite hormone with slight stimulation. Therefore, the abnormal secretion of orexin may be an important mechanism for the occurrence of insomnia in the elderly.

### 3.2. The pathological factors

Not only can aging itself lead to sleep problems, but many of the pathological problems arising from the aging process are also commonly associated with sleep. Older adults frequently experience multiple health conditions, including but not limited to pain syndromes, cardiovascular diseases, urinary tract disorders, and cancer. Each of these conditions can contribute to the development of sleep disorders through specific symptoms associated with the illnesses or anxiety induced by these diseases.^[75]^ It has long been established that mental illnesses significantly elevate the risk of insomnia among the elderly population. Insomnia is a prominent characteristic of numerous mental disorders, and these 2 conditions are intricately linked, exhibiting both causal and consequential relationships.^[76]^

Compared with the single disease impact on sleep problems, higher risk of sleep problems in older people is one of the main problems of the accumulation of comorbidity. More than a quarter of older adults have 2 or more chronic conditions, and the prevalence of multiple chronic conditions increases with age.^[77]^ It has been reported that the proportion of people aged 65 to 74 years suffering from 2 or more chronic diseases is 62%, while the proportion of people aged 85 years and above increases to 82%.^[78]^ In fact, this kind of sick coexistence has become so prevalent in the elderly, the sick coexistence refers to the same person at the same time with 2 or more than 2 kinds of chronic disease, mainly analyzes the number and severity of disease, and considering the body function disorder and the effect of social psychological factors.

With more and more health problems in the elderly, the possibility of insomnia also gradually increases. A survey conducted by the National Sleep Foundation indicates that 36% of older adults report experiencing sleep-related issues.^[79]^ In the population with 3 or fewer comorbidities, this proportion increased to 52%, while in the population with 4 or more comorbidities, it rose to 69%. Known item kind of disease can affect sleep, comorbidity would be compounded the already fragile elderly.

### 3.3. Substance use and substance abuse

Medication use represents a significant factor contributing to the increased risk of insomnia among older adults, who frequently experience multiple health conditions and consequently may require various medications for their treatment. In this demographic, there is a notable rise in the utilization of both prescription medications and dietary supplements. A recent study^[80]^ indicated that 88% of older adults reported using at least one prescription drug, 38% utilized over-the-counter medications, and 64% consumed dietary supplements.

The elderly commonly used different categories of drugs can affect morpheus^[75]^ through a variety of mechanisms. One effect is to cause increased daytime sleepiness, such as with certain antihistamines. Certain medications may exacerbate insomnia. For instance, the use of opioid analgesics has been associated with a worsening of sleep apnea symptoms. Additionally, some beta-blockers have demonstrated an inhibitory effect on melatonin secretion, which can lead to increased sleep fragmentation. Certain medications may adversely affect sleep by exacerbating preexisting conditions or inducing sleep disturbances, such as the diuretic effects that lead to increased nocturia. With the increase of age-related diseases and complications, this situation is more and more common, making the elderly face – drugs and drug interaction – disease risk. The cascade effect refers to the side effects caused by the use of drugs to treat diseases, which may be further complicated by polypharmacy.^[81]^

In addition to drug use, some substance abuse can affect sleep, influential including alcohol, coffee and smoking. Acute alcohol consumption, while potentially reducing sleep latency, is associated with an increased frequency of awakenings and a reduction in overall sleep duration. Furthermore, alcohol intake leads to a decrease in the tone of the pharyngeal muscles, which may exacerbate disorders related to sleep-related breathing.^[82]^ The stimulating effect of caffeine can increase sleep latency and the number of awakenings, resulting in shorter sleep duration.^[83]^ Nicotine in tobacco may promote wakefulness by affecting acetylcholine transmission in the central nervous system, which in turn leads to insomnia.^[84]^

### 3.4. The social psychological factors

Social isolation rate increase after retirement, that is, reduce the contact with people with a lot of old people live alone, there are^[85]^ according to the survey, 28.3% of 65 and older adults living alone. Social isolation can affect sleep by affecting sleep hygiene and zeitgeber. Sleep hygiene encompasses a set of behavioral and environmental practices designed to enhance the quality of sleep. These recommendations include, but are not limited to, the avoidance of caffeine and alcohol consumption, engaging in appropriate physical exercise, and refraining from daytime napping.^[86]^ Zeitgebers are environmental cues that synchronize circadian rhythms to a 24-hour cycle and facilitate regular sleep-wake patterns.^[87]^ While light is the primary zeitgeber, other factors such as physical activity, meal timing, and various social signals also play significant roles. For the elderly social isolation, they may not have full access to the zeitgeber, lead to irregular sleep – wake model. Before the evidence suggests that in the elderly feel social isolation, insomnia and sleepiness report is more, and appropriately increase activity and satisfaction to the social life can protect people and above 65 from insomnia.^[88]^

## 4. Treatment characteristics of chronic insomnia in the elderly

The goal of treatment for chronic insomnia in the elderly is to improve the quality and quantity of sleep, reduce the pain and anxiety caused by poor sleep, and improve daytime function. Aging increases percentage body fat and decreases total body water and plasma proteins, leading to a prolonged elimination half-life and potential risk of adverse effects. Therefore, the elderly should carefully choose drug treatment when choosing treatment methods, and choose non-drug treatment methods first.^[89]^

### 4.1. Non-pharmacological treatment

#### 4.1.1. Cognitive behavioral therapy

Cognitive behavioral therapy designed to correct formation during insomnia or bad behavior, and to promote the development of the idea, is considered the gold standard for treatment of insomnia, its effect is similar to the hypnotic drug or greater than the hypnotic drugs, and unlike hypnotics, cognitive behavioral therapy can still maintain a therapeutic effect after treatment stopped.^[90]^ These effects are in primary insomnia and comorbid insomnia can be found in.^[91]^

Cognitive behavioral therapy consists of 5 main components: stimulus control, sleep restriction, relaxation techniques, cognitive therapy, and education about sleep hygiene.

Stimulus control is a readaptation treatment that forces the distinction between daytime and sleep environments.^[92]^ For poor sleep, the bedroom can let a person associate to awake and awakening, leading to a into the bedroom to sleep instead. Treatment consisted of removing all stimuli that might be incompatible with sleep (reading, television watching, and mobile phone use) and excluding the sleep environment from the living area. If an individual is unable to fall asleep within 20 minutes or experiences awakenings during the night, it is recommended that they rise from bed and refrain from returning until a sense of sleepiness is felt. After a period of treatment, the patient subconsciously separates the living area and the sleeping area, and finally achieves the purpose of sleeping as soon as he returns to the bedroom.

Sleep restriction is to better match the duration of bed rest with the duration of nighttime sleep.^[93]^ The patient maintains a sleep diary to ascertain the average duration of sleep. Subsequently, the time allocated for being in bed is established as the average sleep duration plus an additional 30 minutes, and a consistent wake-up time is determined. This intervention has been shown to effectively reduce the total time spent in bed while providing individuals with a psychological cue that their bed is now associated with safe and restorative sleep.

Relaxation techniques include progressive relaxation, imagery training, biofeedback, meditation, hypnosis, and autotraining, with little evidence of the superiority of any one approach.^[94]^ Muscle tension and heightened brain activity can contribute to the onset of insomnia. Relaxation therapy has been shown to alleviate both physical and mental arousal; however, its efficacy is relatively limited, and it tends to yield better results when integrated with other therapeutic interventions.

Cognitive therapy involves making patients recognize that unhelpful and negative thoughts about sleep increase levels of physical and psychological arousal.^[95]^ Set aside 15 to 20 minutes early before bed to write down anything you have to do for the next day, make plans for the next day, and address any problems that may be affecting your sleep during the night. Psychological hint to oneself “I have solved these problems, now I can sleep with peace of mind,” increase psychological hint, reduce psychological burden, help sleep. Should abandon some bad faith at the same time, such as “if I don’t sleep well tonight, will affect tomorrow’s status,” these beliefs to sleep without benefits, not only will let the brain get excited state, and harder to fall asleep. Another kind of the opposite tack, cognitive therapy is to encourage patients to stay awake, rather than trying to fall asleep, to strengthen sleep driving force, and improve sleep.^[96]^

Sleep health education including several kinds of promoting the healthy and stable and create a sleep environment without interference interventions.^[97]^ These measures include avoiding daytime naps, maintaining a regular sleep schedule, limiting the intake of harmful substances such as caffeine, nicotine, and alcohol, ensuring sleep places are protected from light and noise, and creating a comfortable sleep environment to avoid disrupting sleep.

#### 4.1.2. Bright light exposure

Bright light exposure (natural or artificial) is an important aspect of the treatment of insomnia. Bright light is an effective synchronizer of circadian rhythms in humans. Particularly, exposure to morning light can be integrated with activities such as walking to enhance the quality of nighttime sleep and mitigate sleep inertia experienced in the morning.^[98]^

#### 4.1.3. Noninvasive brain stimulation

noninvasive brain stimulation techniques, such as thermal stimulation and transcranial direct current stimulation, have the potential to enhance sleep quality when used in conjunction with cognitive and pharmacological therapies. These technologies can induce localized alterations in specific regions of the human cerebral cortex, thereby modulating arousal levels and facilitating improved sleep. Studies have shown^[99]^ that in healthy subjects, transcranial direct current stimulation can induce EEG slow waves for a short time and increase slow wave sleep. In patients with insomnia, frontal thermal stimulation dose dependence to improve sleep latency and sleep efficiency.^[100]^

### 4.2. Drug treatment

#### 4.2.1. Benzodiazepine and non-benzodiazepine sedatives

Benzodiazepines and non-benzodiazepines share common mechanisms of action. They act by binding to specific receptor sites on GABA receptors, increasing the intrinsic activity of the inhibitory neurotransmitter GABA and enhancing inhibitory output to all major cell populations in the brainstem and hypothalamus that promote arousal. The difference is that non-benzodiazepines are more selective for α-1 subclass receptors and have mild anxiolytic, amnestic, and anticonvulsant effects while causing sedation compared to benzodiazepines.^[101]^ These 2 kinds of drugs can be effective in the short term treatment of insomnia related parameters, including shortening sleep incubation period began, reducing frequency of nighttime awakening, increase total sleep time and improve sleep quality, but the long-term use of the fiasco is.^[102,103]^

Long-term use of these drugs can lead to tolerance, dependence, rebound insomnia, residual daytime sedation, motor incoordination, cognitive impairment, and an increased risk of stroke.^[104]^ If these drugs are used together, there is an additive effect. As a result of these reactions, should avoid the use of these drugs in the elderly. In 2015 the American geriatrics society is strongly recommended that the elderly avoid the use of these drugs in Beers standard.^[105]^

The pharmacokinetic properties of such drugs determine the differences between the effects of drugs on sleep parameters. For example, zolpideme has a short half-life (2–3 hours), so there is less potential for residual daytime adverse events than zopicone, which has a longer half-life (5–6 hours). However, zolpidem’s short half-life makes it less useful in the treatment of sleep-maintenance insomnia. It has a faster onset of action and therefore can be used to treat narcoleptic insomnia (to reduce the latency of sleep onset).^[106]^

#### 4.2.2. Melatonin receptor agonist

Melatonin receptor agonists are often used in the treatment of senile chronic insomnia, its representative drug for ray for amine. In a study of elderly people (age ≥ 65 years),^[107]^ treatment with ramelteamide significantly shortened sleep latency after 5 weeks without significant rebound insomnia or withdrawal effects, and no dependence, memory impairment, and nocturnal gait instability were found.^[108]^

#### 4.2.3. Antidepressant drugs

A variety of antidepressants, which have sedative properties, can be used to treat insomnia, but some classes are not suitable for the elderly.

Trazodone is widely used in the treatment of insomnia,^[109]^ which has a certain effect in shortening sleep latency and improving sleep efficiency. However, in the elderly population, adverse events such as dizziness, arrhythmia, orthostatic hypotension, and potential priapism are also significant, so it is not recommended for elderly patients.

Mirtazapine, an antidepressant with 5-HT antagonism, also improves insomnia. In a study of 18 to 75-year-old adults according to the results of^[110]^: mirtazapine group at 2 weeks after treatment, the incubation period of sleep, sleep and sleep efficiency were improved significantly after awakening is; However, they should not be used to treat insomnia in the absence of depression because of conflicting evidence and habitual use of their sedative effects.

Of all antidepressants, only doxepin has been approved by the Food and Drug Administration for the treatment of insomnia, which is selective for histamine receptors. Studies using 1 and 3 mg doses in men and women aged 65 years and older have shown^[111]^ that doxepin 1 and 3 mg significantly improved sleep duration, sleep quality, and overall sleep time in subjects.

#### 4.2.4. Polymorphism in the orexin receptor antagonists

Orexin receptor antagonists target arousal-promoting neuropeptides that regulate the sleep-wake cycle. Its representative drug is Suvaresan, which is the first FDA-approved dual orexin receptor antagonist. Demonstrated its efficacy in reducing sleep latency and increasing total sleep time. Suvaresan has been studied in elderly patients (age ≥ 65 years) and found to have significant efficacy and safety.^[112]^ And found that Sue WoLeiSheng shown beneficial effects in delirium,^[113]^ not like other sleeping drugs on memory loss, also can improve the elderly patients with high blood pressure of sleep, and well tolerated.^[114]^

## 5. Summary and outlook

Due to degenerative changes in organs and tissues, insomnia in the elderly shows unique changes in circadian rhythm and sleep structure, and the incidence is increasing in the elderly. Insomnia has developed into one of the most common disorders among the elderly. At the same time, insomnia can induce a variety of chronic diseases and affect the quality of life of patients. Under the background of population aging, healthy aging has become the goal pursued by all walks of life and the elderly themselves. So how to solve the problem of the elderly insomnia, and the influence of the elderly life quality problems caused by insomnia, needs the joint efforts of the society from all walks of life.

The treatment of chronic insomnia disorders including: as for non-drug therapy and drug therapy in 2 ways. Non-drug treatments, have apparent curative effect, and the advantage of no side effects. In particular, cognitive therapy has been recommended as the first-line treatment for chronic insomnia by the European Association of Sleep Physicians. However, such therapies have drawbacks such as long onset time, low patient compliance, and lack of professional clinicians to implement them, so they have not been widely used in the treatment of insomnia. Medications should be carefully selected in the treatment of insomnia in the elderly. Long-term use of hypnotics can lead to tolerance, dependence, rebound insomnia, residual daytime sedation, motor incoordination, cognitive impairment, and increased risk of stroke. Therefore, according to the manifestations, causes and treatment characteristics of chronic insomnia in the elderly, it will be the focus of future research to study a drug or non-drug therapy with good efficacy, high safety and convenient implementation method for the treatment of chronic insomnia in the elderly.

## Author contributions

**Conceptualization:** Jixiang Ren.

**Formal analysis:** LiBo Xia.

**Investigation:** LiBo Xia.

**Methodology:** LiBo Xia.

**Supervision:** HaiYan Liu.

**Visualization:** HaiYan Liu.

**Writing – original draft:** LiBo Xia, HaiYan Liu, Jixiang Ren.

**Writing – review & editing:** LiBo Xia, HaiYan Liu, Jixiang Ren.
